# Supplementary material for: Motor-cognitive analysis of dual task walking in chronic obstructive pulmonary disease patients: An observational study using functional near infrared spectroscopy
Source: PLoS One. 2025 Dec 5;20(12):e0337647. doi: 10.1371/journal.pone.0337647 (PMC12680142; doi:10.1371/journal.pone.0337647)
Supplement: S1 File — https://protocols.io/view/motor-cognitive-analysis-of-dual-task-walking-in-c-d5v6869e.pdf). The protocol outlines detailed procedures to precisely conduct the experiment including a list of guidelines, required materials and safety warnings. (PDF) [file pone.0337647.s001.pdf]

Mar 19, 2025

Motor-Cognitive Analysis of Dual Task Walking in Chronic Obstructive Pulmonary Disease Patients: An Analysis Using Functional Near Infrared Spectroscopy

DOI
dx.doi.org/10.17504/protocols.io.rm7vz6mx2gx1/v1

Ahmed S. Hassan[1], Manjiri Kulkarni[1], Dmitry Rozenberg[2,3], W. Darlene Reid*[1,4,5]

[1]Department of Physical Therapy, University of Toronto, Toronto, ON, Canada;
[2]Toronto General Hospital Research Institute, Ajmera Transplant Center, University Health Network, Toronto, ON, Canada;
[3]Division of Respirology, Temerty Faculty of Medicine, University of Toronto, Toronto, ON, Canada;
[4]Interdepartmental Division of Critical Care Medicine, University of Toronto, Toronto, ON, Canada;
[5]KITE, Toronto Rehabilitation Institute, University Health Network, Toronto, ON, Canada

W. Darlene Reid*: *Corresponding author;

SYED AHMED HASSAN
UNIVERSITY OF TORONTO

Create & collaborate more with a free account

Edit and publish protocols, collaborate in communities, share insights through comments, and track progress with run records.

Create free account

OPEN ACCESS

DOI: dx.doi.org/10.17504/protocols.io.rm7vz6mx2gx1/v1

Protocol Citation: Ahmed S. Hassan, Manjiri Kulkarni, Dmitry Rozenberg, W. Darlene Reid* 2025. Motor-Cognitive Analysis of Dual Task Walking in Chronic Obstructive Pulmonary Disease Patients: An Analysis Using Functional Near Infrared Spectroscopy. **protocols.io https://dx.doi.org/10.17504/protocols.io.rm7vz6mx2gx1/v1**

License: This is an open access protocol distributed under the terms of the **Creative Commons Attribution License**, which permits unrestricted use, distribution, and reproduction in any medium, provided the original author and source are credited

Protocol status: Working
We use this protocol and it's working

Created: March 17, 2025

Last Modified: March 19, 2025

Protocol Integer ID: 124574

Keywords: Chronic obstructive pulmonary disease, Prefrontal cortex, Near-infrared spectroscopy, Gait, cognitive analysis of dual task walking, dual task walking, single task walking, dual task in the copd patient, functional near infrared spectroscopy chronic obstructive pulmonary disease, insights of dual task deficit, dual task deficit, motor task, cognitive task, dual task performance, dual task experimental paradigm, single to dual task performance, single task performance, functional near infrared spectroscopy, dual task, neural correlates of the cognitive demand, dual task cost, physical impairment, consistent with greater cognitive demand, decrement to the single task performance, functional state of patient, physical deficit, cognitive analysis, single task, respective single task, infrared spectroscopy, greater cognitive demand, δo2hb over the right dorsolateral prefrontal cortex, motor function, cognitive demand, copd, impairment, chronic obstructive pulmonary disease patient, neural correlate, right dorsola

Funders Acknowledgements:
Canadian Institutes of Health Research (CIHR)
Grant ID: PJM 179846
Canadian Institutes of Health Research (CIHR)
Grant ID: PJT 183640
DR receives research salary support from Temerty Faculty of Medicine and Sandra Faire and Ivan Fecan Professorship in Rehabilitation Medicine

Disclaimer

DISCLAIMER – FOR INFORMATIONAL PURPOSES ONLY; USE AT YOUR OWN RISK

The protocol content here is for informational purposes only and does not constitute legal, medical, clinical, or safety advice, or otherwise; content added to **protocols.io** is not peer reviewed and may not have undergone a formal approval of any kind. Information presented in this protocol should not substitute for independent professional judgment, advice, diagnosis, or treatment. Any action you take or refrain from taking using or relying upon the information presented here is strictly at your own risk. You agree that neither the Company nor any of the authors, contributors, administrators, or anyone else associated with **protocols.io**, can be held responsible for your use of the information contained in or linked to this protocol or any of our Sites/Apps and Services.

Abstract

Chronic Obstructive Pulmonary Disease (COPD), characterized by airflow limitation, commonly manifests cognitive and physical impairments. Although the interplay of cognitive-motor function is required for many daily activities, these impairments are often managed separately. Our protocol applied a dual task experimental paradigm to assess the interaction and potential decrements of simultaneously performing cognitive and motor tasks. This protocol also utilized functional near infrared spectroscopy (fNIRS) to monitor neural correlates (changes in oxygenated hemoglobin, ΔO_2Hb). Two single tasks and a dual task were applied in random order: (1) single task walking evaluated by OPAL sensors to measure gait speed; (2) a cognitive task of determining the number of 5-letter words accurately spelled backwards; (3) dual task walking combined with spelling words backwards. The decrements of performance were evaluated by examining the differences from single to dual task performance as well as normalizing the decrement to the single task performance by the following equation: Dual task cost = [(dual task – single task)/single task] x 100%. The ΔO_2Hb monitored over the prefrontal cortex (participant's forehead) provided neural correlates of the cognitive demands of the single and dual tasks and sample data are provided. Sample data showed that both a COPD patient and older adult (control) walked slower and spelled fewer words with lower accuracy during dual task walking compared to the respective single task. The ΔO_2Hb over the right dorsolateral prefrontal cortex is consistent with greater cognitive demands of both single tasks and the dual task in the COPD patient compared to the older adult. This protocol may be informative to healthcare researchers evaluating the functional state of patients with COPD who are capable of independent ambulation because it can provide insights of dual task deficits, and consequently, highlight strategies to mitigate cognitive and physical deficits required for daily activities.

Guidelines

1. When recruiting participants for the study, ensure that individuals would be safe to complete the protocol by confirming there are no limitations with independent ambulation due to neurological, musculoskeletal or cardiovascular conditions.
2. Utilizing the American College of Sports Medicine (ACSM) screening questionnaire[10,11] can facilitate screening of those with conditions that may pose increased risk during physical exercise. In addition, administer the Record of Medications and Comorbidities assessment[1-3], International Physical Activity Questionnaire (IPAQ)[4], and Montreal Cognitive Assessment (MoCA)[5] to ensure participant safety and eligibility to participate.
3. The selection and pronunciation of the 5-letter words should be attended to and scrutinized by the research team to minimize their potential misunderstanding. The same researcher should be providing the 5-letter words for all participants such that this aspect of the protocol is standardized. Be sure to avoid homonyms (e.g., plane-plain, brake-break, write-write, steal-steel); avoid palindrome words (e.g. madam, civic); and avoid words that the participant may perceive as an instruction (e.g., again).
4. Limiting the entire duration of the testing session to ~ 01:30:00 is often essential to minimize fatigue during the session and long-lasting fatigue for several days thereafter.
5. Ensure that the testing area is quiet to avoid distractions and/or interference with communication.

Materials

Materials and Equipment:

A	B	C
Name	Company	Catalog Number
fNIRS (frequency: 4.4 Hz) COBI (software) fnirSoft (software)	BIOPAC, 42 Aero Camino, Goleta, CA 93117, USA	FNIR100W-1
Opal APDM System Sensors Mobility Lab Software	APDM Wearable Technologies Inc., 7204 SW Durham Rd #800, Portland, OR 97224 2828 SW Corbett Avenue Portland, OR 97201	
Nonin 9590 Onyx Finger Pulse Oximeter	Performance Health, 6715 Millcreek Drive, Unit 2, Mississauga, ON L5N 5V2	
Handheld Spirometer	Vitalograph Ltd., Ennis Ireland	copd-6TM, model 4000
3M™ Double Stick Discs, 102 each/pack, 6 pack/case, 2181	https://lifesupply.ca/3m-2181-littman-double-stick-discs-pk-102/ or https://www.littmann.com/3M/en_US/p/d/v000094905/	SKU: A3M 2181

Safety warnings

Safety Warnings

- Remove any unnecessary objects around the testing spots to eliminate tripping hazards.
- Application of the fNIRS device needs to be tight enough to minimize movement and eliminate ambient light, but an overly tight application can induce a headache. This concern can be minimized by using medical grade double stick discs applied to each optode site.
- Ensure area is well-lit to enhance visibility and safety during walking.

Ethics statement

This protocol needs prior approval by the users' Institutional Review Board (IRB).

Before start

Before starting the protocol, ensure that research personnel are well positioned around the testing area to minimize any risk of falls in case a participant loses balance.

Demographics and Clinical Characteristics

1 Record participant's age, height, weight, sex, and dominant leg in an anonymized data collection form with participant's ID.

Questionnaires

2 Administer the following questionnaires: Record of Medications and Comorbidities form[1-3], International Physical Activity Questionnaire (IPAQ)[4], and Montreal Cognitive Assessment (MoCA)[5] (https://mocacognition.com/).

- *Participant can take a break here before proceeding with data collection using the functional Near Infrared Spectroscopy (fNIRS) device and OPAL sensors (gait speed can also be assessed using other modalities e.g., Zeno Walkway). Once the fNIRS is connected, it is more difficult to provide a break.*

Mounting Wireless fNIRS Over the Prefrontal Cortex and Connecting Device to Software

3 Pin hair away from forehead, if required, and clean forehead with an alcohol swab. Let air dry.

4 Place the portable, non-invasive fNIRS device (Fig. 1) following the International 10-20 system of EEG (Electroencephalography) over the PFC[6,7].

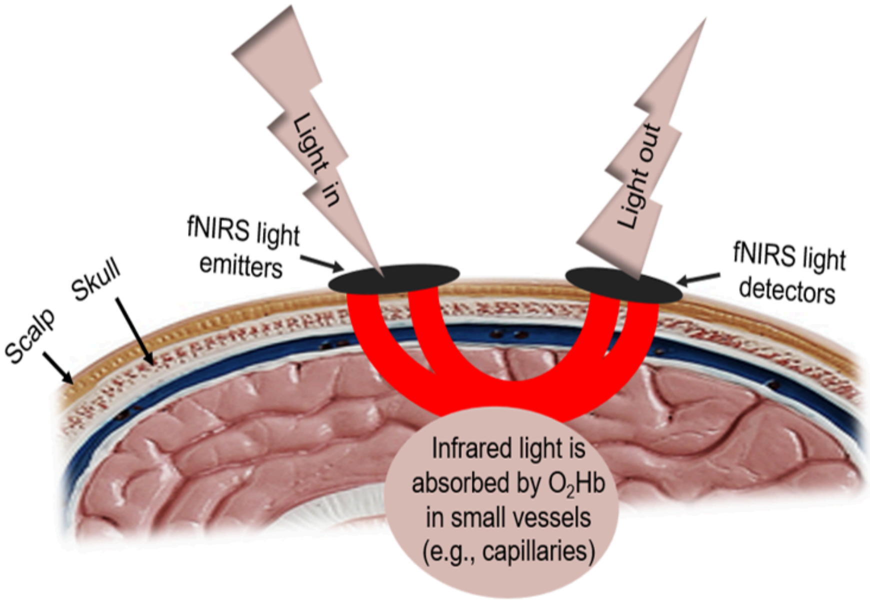

Figure 1: Principles of measurement by functional near infrared spectroscopy (fNIRS). The fNIRS device is applied over the cortical regions of interest. Infrared light emitted from the device is transmitted through the scalp, skull and to the small blood vessels of the underlying brain tissue, where it is absorbed by oxygenated (O_2Hb) and deoxygenated hemoglobin (HHb). The fNIRS light detectors measure infrared light that is transmitted back to estimate the relative change of O_2Hb and HHb in the underlying tissue. With continuous wave fNIRS, only the changes of these outcomes are estimated. Moreover, with continuous wave fNIRS, the depth of penetration of the infrared light is considered to be one-half of the interoptode distance. Part of this figure (brain) is from ID15062121© Springdt313 | Dreamstime.com

5 Secure the fNIRS device at the back of the head.

Note

Be sure that the fNIRS device is not too tight or it may induce a headache.

6 Place a black cloth band over the device to avoid the added input of ambient light from contaminating and altering the fNIRS output.

7 Connect the fNIRS device with the software according to the requirements of the particular program[8].

8 Click on “Start Current Device” under “Device” in the left panel and ensure appropriate signal is being received (between 1000mV and 3500mV) from the device. If the light signal is outside these bounds, adjust the detector gain (minimum 1) and then LED Light (minimum 5) settings[9].

Attach the Device to Measure Gait Speed

9 This device consists of OPAL wearable sensors, a docking system, an access point to enable wireless streaming and software:

Participants are connected to three OPAL sensors (Fig. 2)- attach with adjustable Velcro straps: (i) right foot (centered on the top of the foot/shoe/sock - port faces outwards); (ii) left foot (centered on the top of the foot/shoe/sock- port faces outwards); (iii) just below the waist at the level of the second sacral spinous process (S2). S2 is at the level of the posterior superior iliac spine. Ensure the port is facing downwards.

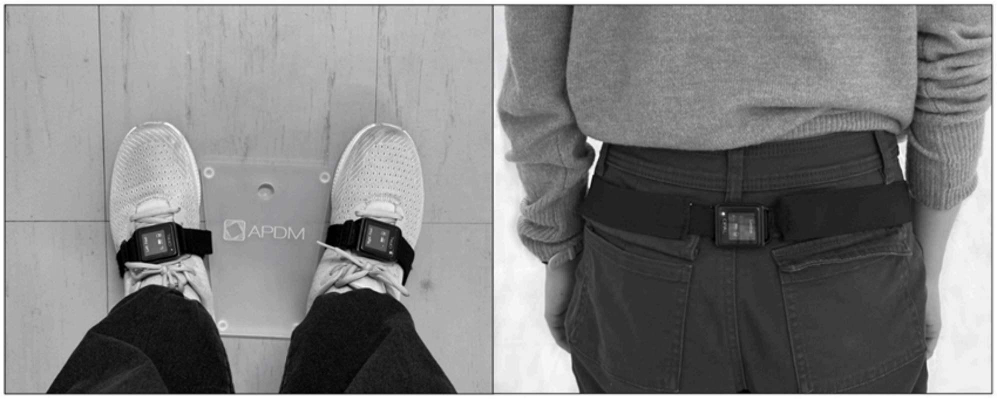

Figure 2: The wearable sensors to measure gait speed are attached to the right and left foot (each centered on the top of the foot) (left panel) and in the midline just below the waist at the level of the second sacral spinous process (S2) (right panel). S2 is at the level of the posterior superior iliac spine. Ensure the port is facing downwards. The starting position of the feet is standardized by positioning them adjacent to the docking system.

10 On the software, select “New Subject” and enter participant number. A folder with the patient number will be created.

11 Click on the folder and go on “Test Selection”. Then click on the drop-down menu next to “Walk” and select “Open ended”. Click on the “+” sign next to it and it appears on the right side under “selected tests”.

12 After asking the participant to stand at the starting point, place the footplate provided with the device in between the feet to standardize the feet distance.

13 After explaining the walk test (details below) and when researchers and participant are in position, click on "start", and then the device beeps with a countdown from 3, and the participant is instructed to "Go" to begin with the walk test.

14 Click "stop" when participant completes the walk and crosses the finish point.

Single and Dual Tasks (Randomized Order) (Fig. 3) - Baseline task

1m

15 Hand the participant a deck of flash cards that have short easy words and matching pictures (you may use a cut-off of less than 6 letters and words that readily match the picture).

16 Ask the participant to spell the words written on the cards in their head at a relaxing speed. Also inform the participant that it does not matter how many cards they spell forwards and this task is to facilitate a baseline resting task.

17 Instruct the participant that they will do this task for 00:01:00 . 1m

Note

Perform this task before and after every single and dual task.

Single and Dual Tasks (Randomized Order) (Fig. 3) - Single task – walking at preferred pace (PPW)

1m

18 In a quiet room or hallway, set up a course of 5 or 10 meters demarcated at either end by tape on the floor or cones with a chair at both ends for the participant to rest if required.

19 Have a member of the research team demonstrate walking the course and turning around the cones at each end of the course.

20 Instruct the participant to stand upright and look straight ahead at a marked position at head-height on the opposite wall.

21 Instruct the participant that upon a countdown of "3-2-1" followed by "Go", to walk in a straight line at the preferred or usual pace to the second marked position (5-10 meters away, depending on the hallway/room available), to turn around the cone and return to the starting position. The participant should proceed for a total of 30 meters.

Single and Dual tasks (randomized order) (Fig. 3) - Single task – spelling backwards (SB)

1m

22 Have a prepared list of 5 letter words that can be clearly articulated (Appendix).

23 Instruct the participant that you will be saying 5 letter words, which they need to spell backwards. Provide the participant an example of spelling a word backwards for familiarization (e.g., "paint" → "T" "N" "I" "A" "P").

24 Once the participant is familiar with the task of spelling words backwards, begin stating the first word and wait for a response, then repeat this cycle for a total time of 00:01:00 . Record the accuracy of the responses. One point is allocated for each word spelled backwards accurately.

1m

Single and Dual tasks (randomized order) (Fig. 3) - Dual task – spelling backwards while walking at preferred pace

1m

25 Instruct the participant that "*Now I will ask you to do two things at the same time. I will be asking you to spell words backwards while you are walking at your usual or preferred pace.*"

- Repeat the same instructions of PPW and SB stated above to test the participant performing both tasks simultaneously.

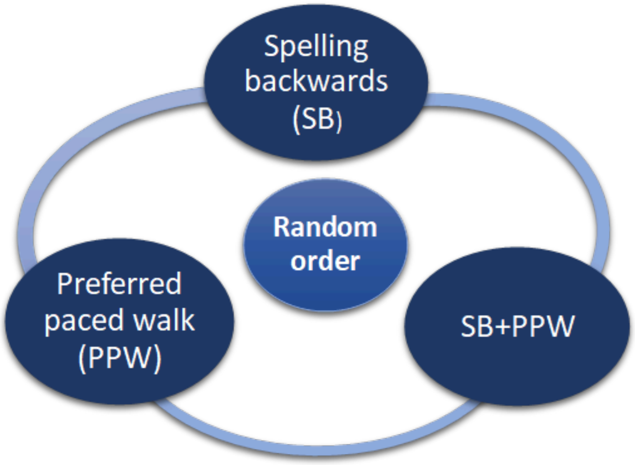

Figure 3: Single and dual tasks are applied in random order and included: spelling 5-letter words backwards (SB), 30m preferred paced walk (PPW) and dual task SB+PPW.

Fig. 3

Data Processing

26 **Refining data and calculating oxygenation (O_2Hb) values**

- Load the light data collected in the data acquisition software (Cognitive Optical Brain Imaging [COBI]) into the processing software (fNIRSoft). Next, remove motion artefacts and address physiological artefacts by applying filter of 57 hamming order and 0.05 cut off frequency.

27 Define and save the single and dual tasks' starting and end times for extraction of oxygenation data converted from the processed light data in previous step.

28 The saved time periods will open up in "fnirSoft Processing Tool". Under "Input Variables" choose the light graph block files and leave the "baseline variable(s)" section blank to use the first 20 data points of each block as a baseline against which the oxygenation changes will be measured.

29 Click "Execute". Under "Export" tab, export the processed files, now containing O_2Hb data as "Excel" files for statistical analysis.

Appendix - Sets of 5 Letter Words to Spell Backwards.

30

Note

Efforts have been made to exclude homonyms and palindromes

Set 1

EARTH<br>E() A() R() T() H()	CURVE<br>C() U() R() V() E()	GUEST<br>G() U() E() S() T()	ABOUT<br>A() B() O() U() T()	BUILT<br>B() U() I() L() T()
MONTH<br>M() O() N() T() H()	HOUSE<br>H() O() U() S() E()	TOUCH<br>T() O() U() C() H()	LEARN<br>L() E() A() R() N()	DOZEN<br>D() O() Z() E() N()
COUNT<br>C() O() U() N() T()	ABOVE<br>A() B() O() V() E()	BASIC<br>B() A() S() I() C()	STYLE<br>S() T() Y() L() E()	SOUND<br>S() O() U() N() D()
WATCH<br>W() A() T() C() H()	EARLY<br>E() A() R() L() Y()	CABLE<br>C() A() B() L() E()	SCALE<br>S() C() A() L() E()	SPEED<br>S() P() E() E() D()
NORTH<br>N() O() R() T() H()	SOUTH<br>S() O() U() T() H()	LOCAL<br>L() O() C() A() L()	CHAIN<br>C() H() A() I() N()	SMOKE<br>S() M() O() K() E()

Set 2

HEAVY<br>H() E() A() V() Y()	LUNCH<br>L() U() N() C() H()	MOUSE<br>M() O() U() S() E()	SEVEN<br>S() E() V() E() N()	CHEAP<br>C() H() E() A() P()
SENSE<br>S() E() N() S() E()	FAITH<br>F() A() I() T() H()	APPLE<br>A() P() P() L() E()	LARGE<br>L() A() R() G() E()	TEACH<br>T() E() A() C() H()
UPSET<br>U() P() S() E() T()	ALERT<br>A() L() E() R() T()	TIRED<br>T() I() R() E() D()	MONEY<br>M() O() N() E() Y()	ADULT<br>A() D() U() L() T()
SHIRT<br>S() H() I() R() T()	ISSUE<br>I() S() S() U() E()	MAGIC<br>M() A() G() I() C()	MOVIE<br>M() O() V() I() E()	SOLVE<br>S() O() L() V() E()
SWEET<br>S() W() E() E() T()	ADMIT<br>A() D() M() I() T()	CATCH<br>C() A() T() C() H()	ALONE<br>A() L() O() N() E()	BLACK<br>B() L() A() C() K()

Set 3

SMALL<br>S() M() A() L() L()	WASTE<br>W() A() S() T() E()	QUICK<br>Q() U() I() C() K()	PAINT<br>P() A() I() N() T()	TOPIC<br>T() O() P() I() C()
MOUTH<br>M() O() U() T() H()	SHELF<br>S() H() E() L() F()	TASTE<br>T() A() S() T() E()	CRAFT<br>C() R() A() F() T()	SLEEP<br>S() L() E() E() P()
HAPPY<br>H() A() P() P() Y()	BREAD<br>B() R() E() A() D()	EQUAL<br>E() Q() U() A() L()	BLIND<br>B() L() I() N() D()	SHAPE<br>S() H() A() P() E()
DANCE<br>D() A() N() C() E()	RADIO<br>R() A() D() I() O()	SHIFT<br>S() H() I() F() T()	LIGHT<br>L() I() G() H() T()	ACTOR<br>A() C() T() O() R()
EMPTY<br>E() M() P() T() Y()	CHEST<br>C() H() E() S() T()	TABLE<br>T() A() B() L() E()	FINAL<br>F() I() N() A() L()	HORSE<br>H() O() R() S() E()

Set 4

THINK<br>T() H() I() N() K()	HUMAN<br>H() U() M() A() N()	GIANT<br>G() I() A() N() T()	NIGHT<br>N() I() G() H() T()	FIRST<br>F() I() R() S() T()
FIFTY<br>F() I() F() T() Y()	BLOOD<br>B() L() O() O() D()	NURSE<br>N() U() R() S() E()	BEACH<br>B() E() A() C() H()	ANGLE<br>A() N() G() L() E()
TEXAS<br>T() E() X() A() S()	BELOW<br>B() E() L() O() W()	FORCE<br>F() O() R() C() E()	BROKE<br>B() R() O() K() E()	SHOCK<br>S() H() O() C() K()
SPACE<br>S() P() A() C() E()	THEFT<br>T() H() E() F() T()	CYCLE<br>C() Y() C() L() E()	SIXTY<br>S() I() X() T() Y()	STONE<br>S() T() O() N() E()
MARCH<br>M() A() R() C() H()	MOUNT<br>M() O() U() N() T()	LASER<br>L() A() S() E() R()	MUSIC<br>M() U() S() I() C()	LUCKY<br>L() U() C() K() Y()

Protocol references

Citations relevant to protocol steps

1.

Citation

Hassan SA, Bonetti LV, Kasawara KT, Beal DS, Rozenberg D, Reid WD (2022)
. Decreased automaticity contributes to dual task decrements in older compared to younger adults..

https://doi.org/10.1007/s00421-022-04891-w LINK

2.

Citation

Hassan SA, Bonetti LV, Kasawara KT, Stanbrook MB, Rozenberg D, Reid WD (2022)
. Loss of Neural Automaticity Contributes to Slower Walking in COPD Patients..

https://doi.org/10.3390/cells11101606 LINK

3.

Citation

Hassan SA, Campos MA, Kasawara KT, Bonetti LV, Patterson KK, Beal DS, Fregonezi GAF, Stanbrook MB, Reid WD (2020)
. Changes in Oxyhemoglobin Concentration in the Prefrontal Cortex during Cognitive-Motor Dual Tasks in People with Chronic Obstructive Pulmonary Disease..

https://doi.org/10.1080/15412555.2020.1767561 LINK

Additional Notes

For detailed use of the software used in data collection and processing, please see

- https://www.biopac.com/wp-content/uploads/fnirsoft-user-manual.pdf
- https://www.biopac.com/wp-content/uploads/fnir-cobi-manual.pdf

References

1. Charlson, M. E., Pompei, P., Ales, K. L. & MacKenzie, C. R. A new method of classifying prognostic comorbidity in longitudinal studies: Development and validation. J. Chronic Dis. **40**, 373–383 (1987).
2. Deyo, R. A., Cherkin, D. C. & Ciol, M. A. Adapting a clinical comorbidity index for use with ICD-9-CM administrative databases. J. Clin. Epidemiol. **45**, 613–619 (1992).
3. HajGhanbari, B., Yamabayashi, C., Garland, R., Road, J. & Reid, W. D. The Relationship Between Pain and Comorbid Health Conditions in People with Chronic Obstructive Pulmonary Disease. Cardiopulm. Phys. Ther. J. **25**, 29–35 (2014).
4. Craig, C. L. et al. International Physical Activity Questionnaire: 12-Country Reliability and Validity. Med. Sci. Sports Exerc. **35**, 1381 (2003).
5. Nasreddine, Z. S. et al. The Montreal Cognitive Assessment, MoCA: A Brief Screening Tool For Mild Cognitive Impairment. J. Am. Geriatr. Soc. **53**, 695–699 (2005).
6. McKendrick, R., Mehta, R., Ayaz, H., Scheldrup, M. & Parasuraman, R. Prefrontal Hemodynamics of Physical Activity and Environmental Complexity During Cognitive Work. Hum. Factors **59**, 147–162 (2017).
7. McKendrick, R. et al. Into the Wild: Neuroergonomic Differentiation of Hand-Held and Augmented Reality Wearable Displays during Outdoor Navigation with Functional Near Infrared Spectroscopy. Front. Hum. Neurosci. **10**, (2016).
8. Ayaz, H. 'Functional Near Infrared Spectroscopy based Brain Computer Interface.' PhD Thesis, Drexel University, Philadelphia, PA. (2010).
9. Ayaz, H. Analytical Software and Stimulus-Presentation Platform to Utilize, Visualize and Analyze Near-infrared Spectroscopy Measures. (Drexel University, United States -- Pennsylvania, 2005).
10. Thompson, W. R., Gordon, N. F. & Pescatello, L. S. ACSM's Guidelines for Exercise Testing and Prescription. (Wolters Kluwer/Lippincott Williams & Wilkins, Philadelphia, 2010).
11. AHA/ACSM Joint Position Statement: Recommendations for Cardiovascular Screening, Staffing, and Emergency Policies at Health/Fitness Facilities. Med. Sci. Sports Exerc. **30**, 1009 (1998).

Citations

Hassan SA, Bonetti LV, Kasawara KT, Beal DS, Rozenberg D, Reid WD. Decreased automaticity contributes to dual task decrements in older compared to younger adults.
https://doi.org/10.1007/s00421-022-04891-w

Hassan SA, Bonetti LV, Kasawara KT, Stanbrook MB, Rozenberg D, Reid WD. Loss of Neural Automaticity Contributes to Slower Walking in COPD Patients.
https://doi.org/10.3390/cells11101606

Hassan SA, Campos MA, Kasawara KT, Bonetti LV, Patterson KK, Beal DS, Fregonezi GAF, Stanbrook MB, Reid WD. Changes in Oxyhemoglobin Concentration in the Prefrontal Cortex during Cognitive-Motor Dual Tasks in People with Chronic Obstructive Pulmonary Disease.
https://doi.org/10.1080/15412555.2020.1767561
