# Supplementary material for: Motor-cognitive analysis of dual task walking in chronic obstructive pulmonary disease patients: An observational study using functional near infrared spectroscopy
Source: PLoS One. 2025 Dec 5;20(12):e0337647. doi: 10.1371/journal.pone.0337647 (PMC12680142; doi:10.1371/journal.pone.0337647)
Supplement: Attachment — Submitted filename: Dual_Task_Hassan_SA_Response_to_Reviewers.docx [file pone.0337647.s002.docx]

**POINT BY POINT RESPONSE to manuscript PONE-D-25-15334R1**

Motor-Cognitive Analysis of Dual task Walking in Chronic Obstructive Pulmonary Disease Patients: An analysis using functional Near Infrared Spectroscopy

**1.** We noted in your submission details that a portion of your manuscript may have been presented or published elsewhere:

“None of the results data or figures were published elsewhere; however, aggregate data from which a sample of data was taken has been published:

Hassan SA, Bonetti LV, Kasawara KT, Stanbrook MB, Rozenberg D, Reid WD. Loss of Neural Automaticity Contributes to Slower Walking in COPD Patients. Cells. 2022 May 11;11(10):1606.

This has been appropriately cited with in the article text.”

Please clarify whether this [conference proceeding or publication] was peer-reviewed and formally published. If this work was previously peer-reviewed and published, in the cover letter please provide the reason that this work does not constitute dual publication and should be included in the current manuscript.

**Response**: An explanation for this comment was provided in the previous submission dated September 20, 2025, as an attachment as well as in the comment box “Response to Reviewers” For your convenience it is restated below verbatim.

Previous conference proceedings and publications focused on aggregate data from this novel approach to examine dual task walking outcomes in COPD patients compared to healthy individuals. In contrast, the purpose of the current paper is to provide detailed methodology for a dual tasking paradigm that can be used in subsequent research studies and applied clinically. As shown in the methodology (step-by-step protocol: dx.doi.org/10.17504/protocols.io.rm7vz6mx2gx1/v1), in addition to brief descriptions of how to characterize participants, step by step details are provided on how to:

(1) apply the fNIRS device over the prefrontal cortex;

(2) evaluate gait speed;

(3) instruct single and dual tasks

(4) analyse the acquired gait speed, spelling backwards and fNIRS data.

These details including the figures that illustrate these methods have not been provided in previous publications.

Fig 1 of this submission provides examples of the functional near infrared spectroscopy data from two individuals and has not been previously published. Only aggregate data had been previously published.

**2.** We note you have not yet provided a protocols.io PDF version of your protocol and/or a protocols.io DOI. When you submit your revision, please provide a PDF version of your protocol as generated by protocols.io (the file will have the protocols.io logo in the upper right corner of the first page) as a Supporting Information file. The filename should be S1_file.pdf, and you should enter “S1 File” into the Description field. Any additional protocols should be numbered S2, S3, and so on. Please also follow the instructions for Supporting Information captions [https://journals.plos.org/plosone/s/supporting-information#loc-captions]. The title in the caption should read: “Step-by-step protocol, also available on protocols.io.”

Please assign your protocol a protocols.io DOI, if you have not already done so, and include the following line in the Materials and Methods section of your manuscript: “The protocol described in this peer-reviewed article is published on protocols.io (https://dx.doi.org/10.17504/protocols.io.[...]) and is included for printing purposes as S1 File.” You should also supply the DOI in the Protocols.io DOI field of the submission form when you submit your revision.

If you have not yet uploaded your protocol to protocols.io, you are invited to use the platform’s protocol entry service [https://www.protocols.io/we-enter-protocols] for doing so, at no charge. Through this service, the team at protocols.io will enter your protocol for you and format it in a way that takes advantage of the platform’s features. When submitting your protocol to the protocol entry service please include the customer code PLOS2022 in the Note field and indicate that your protocol is associated with a PLOS ONE Lab Protocol Submission. You should also include the title and manuscript number of your PLOS ONE submission.

**Response**: This comment was also addressed in the previous submission dated September 20, 2025 in the updated manuscript file as well as the “Response to Reviewers”. For your convenience it is restated below verbatim.

Protocols.io PDF version was attached with the revised submission and the following statement with protocols.io DOI is also included under Methods section:

- “The protocol described in this peer-reviewed article is published on protocols.io, <https://dx.doi.org/10.17504/protocols.io.rm7vz6mx2gx1/v1> and is included for printing purposes as S1 Protocol.”

**3.** Please amend the title either on the online submission form or in your so that they are identical.

**Response**: We have verified, and the titles are now identical in the online submission form and in the manuscript.

**4.** We note your Data Availability statement as follows:

"All relevant data are within the manuscript and its Supporting Information files.

"All data described in the manuscript is provided in Fig 1 and Table 1. The ΔO2Hb data was collected at a frequency of 4.4Hz using a functional near infrared spectroscopy device, which can be obtained from the scatter plot in Fig 1. Providing any additional participant data on other participants or more particulars on these two individuals would breach compliance with the protocol approved by the University of Toronto’s research ethics board.""

In this instance it seems there may be acceptable restrictions in place that prevent the public sharing of your minimal data. PLOS requires that the minimal data for your study be made available upon request in this case. However, in line with our goal of ensuring long-term data availability to all interested researchers, PLOS’ Data Policy states that authors cannot be the sole named individuals responsible for ensuring data access (http://journals.plos.org/plosone/s/data-availability#loc-acceptable-data-sharing-methods).

Data requests to a non-author institutional point of contact, such as a data access or ethics committee, helps guarantee long term stability and availability of data. Providing interested researchers with a durable point of contact ensures data will be accessible even if an author changes email addresses, institutions, or becomes unavailable to answer requests.

Before we proceed with your manuscript, please address the following prompts:

If there are ethical or legal restrictions on sharing a de-identified data set, please explain the restrictions in detail (e.g., data contain potentially identifying or sensitive patient information) and who has imposed them (e.g., a Research Ethics Committee or Institutional Review Board, etc.). Please also provide non-author contact information (phone/email/hyperlink) for a data access committee, ethics committee, or other institutional body to which data requests may be sent. If applicable, please also provide any necessary information which interested researchers would need when requesting access to data in order to obtain the minimal data set for your study.

**Response**: All data described in the manuscript is provided in Fig 1 and Table 1. The ΔO_2_Hb data was collected at a frequency of 4.4Hz using a functional near infrared spectroscopy device, which can be obtained from the scatter plot in Fig 1. Providing any additional participant data on other participants or more particulars on these two individuals would breach compliance with the protocol approved by the University of Toronto’s research ethics board.

Data are available from the University of Toronto Human Research & Ethics Unit (HREU), Research Oversight & Compliance Office (ROCO) (contact via email: ethics.review@utoronto.ca) for researchers who meet the criteria for access to confidential data.
